# Translation, Adaptation, and Validation of a Korean Version of the Smoking Media Literacy Scale in Adolescents

**DOI:** 10.1111/jspn.70019

**Published:** 2026-07-28

**Authors:** Sunhee Park

**Affiliations:** ^1^ College of Nursing Science, Graduate School of Nursing, East‐West Nursing Research Institute Kyung Hee University Seoul Republic of Korea

**Keywords:** adolescent, pro‐smoking media message, scale validation, smoking, smoking media literacy

## Abstract

**Purpose:**

The enhancement of smoking media literacy (SML) is a crucial strategy to mitigate the adverse effects of pro‐smoking media messages on adolescents. However, the availability of reliable and valid Korean‐language instruments for assessing SML remains limited. Thus, this study aimed to (a) translate and culturally adapt the SML Scale originally developed in English and (b) validate its Korean version among Korean adolescents (*N* = 638 middle and high schoolers).

**Design and Methods:**

The process involved (a) developing a pre‐final Korean version (PFKV) through translation and cultural adaptation, (b) pilot‐testing the PFKV via cognitive debriefing, assessment of translation sufficiency, and content validity testing, (c) conducting exploratory and confirmatory factor analyses, and (d) testing psychometric properties.

**Results:**

The Korean version of the smoking media literacy scale (K‐SMLS) had a two‐factor structure, which was consistent with the original scale's theoretical framework. The K‐SMLS was highly reliable, showing Cronbach's alpha values exceeding 0.8 for the overall scale and its subscales. It also demonstrated satisfactory convergent validity with media literacy and critical thinking disposition, and concurrent validity with current smoking and smoking normative beliefs. These relationships were statistically significant and in the expected directions, consistent with those found in the literature.

**Practice Implications:**

The use of K‐SMLS can contribute to identifying adolescents at risk from pro‐smoking media messages, providing targeted educational interventions, facilitating comparisons of intervention effects across studies and the drawing of informed conclusions, and developing strategies to enhance SML.

## What is Currently Known?

Existing literature highlights that higher levels of smoking media literacy (SML) can effectively reduce the adverse effects of pro‐smoking media messages on smoking risk among adolescents. Despite its significance, reliable and valid instruments to assess SML remain limited. For example, existing scales have not been extensively validated across different cultures, which poses a challenge for the reliable and accurate measurement of SML in diverse populations. The limited availability of psychometrically reliable and valid instruments has restricted both (a) a comprehensive understanding of SML and (b) the evaluation of intervention effects aimed at enhancing SML in adolescents.

## What Does This Article Add?

This article may contribute to research on adolescent smoking by enabling more accurate measurement of SML among Korean adolescents. In this study, the K‐SMLS was found to be a reliable and valid scale based on exploratory and confirmatory factor analyses, as well as psychometric evaluation. This study could contribute to (a) addressing a significant knowledge gap in the psychometric evaluation of SML scales and (b) facilitating efforts to protect Korean adolescents from pro‐smoking messages. Specifically, the implementation of this scale may enable nurses to (a) more accurately identify adolescents who are more susceptible to pro‐smoking media messages and (b) compare the effects of SML interventions among these adolescents, which ultimately could contribute to developing more effective public health strategies for adolescent smoking prevention.

## 1 Introduction

Adolescent smoking has long been an important health issue due to its adverse effects during this stage of life (Benjamin 2012; Kim and Kwon 2019). Nevertheless, approximately 6% of adolescents worldwide were current cigarette smokers (World Health Organization 2021). Furthermore, electronic cigarette use has been rapidly rising in this population worldwide (World Health Organization 2023). In South Korea, 3.60%, 3.00%, and 1.90% of adolescents reported past 30‐day use of cigarettes, electronic cigarettes, and heated tobacco products, respectively (Korea Disease Control and Prevention Agency 2024). Diverse factors, including peer influences, family characteristics, and regulatory environments, have strongly affected adolescent smoking (Gunter et al. 2020; Kim and Lee 2025). While active strategies targeting these factors have been implemented to address this issue, adolescent smoking still remains a serious health concern (Kim and Lee 2025). Thus, additional strategies are needed to more effectively tackle adolescent smoking.

Exposure to pro‐smoking media messages (PSMM) and their influence on adolescent smoking warrant particular attention as an additional intervention target, given that adolescents exposed to PSMM are at an increased risk of smoking (U.S. Department of Health and Human Services 2012). Specifically, the super‐peer theory posits that media messages exert influence on adolescents in a manner similar to—and potentially more powerful than—that of peers, and as a result, health risk behaviors tend to be normalized among adolescents (Arnett 2007). For example, repeated exposure to PSMM leads adolescents to perceive smoking as prevalent and socially acceptable, which increases their smoking risk (Arnett 2007).

Of particular concern is the substantial exposure to PSMM consistently reported in previous U.S. and Korean studies (Agaku et al. 2014; Hébert et al. 2017). This exposure could be especially pronounced in Korea for three reasons. First, approximately 100% of Korean adolescents owned smartphones and spent about 6 h per day on digital devices (Bae et al. 2021). Second, more than half of the Korean media content analyzed contained tobacco products or smoking scenes (National Tobacco Control Center 2019). Lastly, both the number of movies containing tobacco scenes and the number of tobacco depictions per movie were significantly higher in Korea than in other countries (National Tobacco Control Center 2019).

The literature indicates that an increase in media literacy effectively mitigates the negative effects of media content on adolescents (Brown 2006; Committee on Public Education 1999). ML is defined as “the ability of a citizen to access, analyze, and produce information for specific outcomes (Aufderheide and Firestone 1993, p. 6).” Higher ML enables adolescents to think more critically about media messages and gain greater control over mass media influences, which leads to their protection from the potentially harmful effects of these messages (De Abreu et al. 2017; Potter 2010). Given this, an increase in smoking media literacy (SML) can serve as an effective strategy to mitigate the adverse influences of PSMM (De Abreu et al. 2017). To effectively implement strategies to enhance SML, it is crucial to first conduct a rigorous evaluation of SML interventions (Aufderheide and Firestone 1993; Brown 2006; Kubey 1998). This evaluation process necessitates the use of valid and reliable measures to assess SML and accurately understand this phenomenon (Martens 2010; Mellinger and Hanson 2017).

The need for reliable and valid SML assessment instruments is particularly urgent for Korean adolescents, who, as previously noted, face unique challenges due to their high exposure to PSMM. Despite this fact, research on SML in Korea is still in its early stages. For example, only one SML scale exists for Korean adolescents, and this scale may not accurately assess SML levels due to the following major methodological limitations (Kim et al. 2021). The limitations include (a) the absence of cognitive debriefing among Korean adolescents, (b) low factor loadings in exploratory factor analysis, (c) the use of the same sample for both exploratory and confirmatory factor analyses, and (d) the lack of further validation of the developed scale.

Currently, the literature features only one adolescent SML scale developed with a theoretical foundation. This scale was modified and expanded from a validated scale on a theoretical basis, but requires further psychometric evaluation (Primack et al. 2014; Primack et al. 2006). This highlights a significant gap in SML measures, which underscores the importance of evaluating their psychometric properties (Martens 2010; Ptaszek 2019). To address this gap and improve the assessment of SML among Korean adolescents, this study aimed to (a) translate and culturally adapt the SML Scale (SMLS) originally developed in English and (b) validate its Korean version among Korean adolescents. The first step of this study involved translating and adapting the 22‐item English‐language SMLS into a Korean version (Primack et al. 2014). The next step involved validating the Korean version of the smoking media literacy scale (K‐SMLS) using rigorous research methods to address the shortcomings of previous studies (Kim et al. 2021; Primack et al. 2014; Primack et al. 2006).

## 2 Methods

### 2.1 Participants

This study was approved by the Institutional Review Board (KHSIRB‐17‐066‐1[RA]). Using a convenience sampling method, this study selected 650 students attending three middle schools and six high schools in Seoul City, Gyeongsang Province, and Gyeonggi Province in the Republic of Korea. Efforts were made to obtain a reasonably heterogeneous sample across sex, school year (grades 1–3 within each school type), and school type (middle school, vocational high school, and academic high school). Prior to data collection, approval was obtained from the principals of all schools. Written informed consent was also obtained from both participants and their guardians after explaining the purposes of this study. Inclusion criteria were (a) current enrollment in a middle or high school in South Korea and (b) provision of consent by both participants and guardians. Exclusion criteria were (a) communication impairments and (b) an inability to complete the questionnaire. Data were collected over approximately 1 month, from October to November 2017.

This study analyzed data obtained from 638 students after excluding 12 participants due to substantial missing information. Approximately 50% (*n* = 321) of the participants were male, and the mean age was 15.55 years. About 64% (*n* = 408) reported receiving less than 30,000 Korean won (approximately 22.54 U.S. dollars) as average weekly pocket money (Exchange‐Rates n.d.). Additionally, about 86% (*n* = 529) were lifetime non‐smokers, while the remaining participants (*n* = 90) were either former or current smokers. The proportion of lifetime non‐smokers in this sample was consistent with the rate reported in a nationally representative sample of Korean adolescents (86.30%; Korea Disease Control and Prevention Agency 2017).

### 2.2 Measures

This study evaluated the psychometric properties of the K‐SMLS using two scales measuring constructs similar to SML (i.e., media literacy and critical thinking disposition) and three smoking characteristics (i.e., current smoking and two types of smoking normative beliefs).

#### 2.2.1 Smoking Media Literacy

The 17‐item K‐SMLS was developed based on the original English‐language SMLS (Primack et al. 2014). The detailed processes for the K‐SMLS development, including translation and cultural adaptation, were presented in the Procedures section. The original SMLS was a 22‐item, self‐administered scale assessing SML levels among U.S. adolescents (Primack et al. 2014). This scale was modified and expanded from the validated 18‐item version developed in 2006 based on theoretical grounds (Primack et al. 2006). Specifically, Primack and colleagues initially generated 120 items representing eight core concepts of the media literacy framework and finalized the items of the SMLS through psychometric evaluation (Primack et al. 2006). It consisted of two subscales: 11 items for smoking‐specific media literacy (SSML) and 11 items for general media literacy (GML) (Primack et al. 2014). Each subscale included items representing all theoretical core concepts of media literacy: (a) authors and audiences (AA; understanding who creates media and for whom), (b) messages and meanings (MM; understanding how media messages are constructed and interpreted), and (c) representation and reality (RR; understanding how media portrayals relate to or differ from actual reality; Primack et al. 2014; Primack et al. 2006). Each item was measured on a 4‐point Likert scale (0 = strongly disagree, 1 = disagree, 2 = agree, 3 = strongly agree), and a total score was calculated by summing all item responses. These summated scores were then converted to a 10‐point scale (i.e., raw score/maximum possible score × 10) for ease of interpretation and application (Primack et al. 2006). Cronbach's alpha coefficients for both subscales exceeded 0.75, indicating satisfactory reliability (Primack et al. 2014).

#### 2.2.2 Media Literacy

This study used a 17‐item subscale of the Korean media literacy scale to measure the understanding of media characteristics, the correct use of information conveyed by the media, and the critical understanding of media messages (Gwon 2017). Participants were asked to rate their agreement levels with each item on a 5‐point Likert scale (1 = totally disagree, 2 = disagree, 3 = neutral, 4 = agree, 5 = totally agree). The average scores of the items were calculated, and higher scores represented greater levels of media literacy (Cronbach's alpha coefficient = 0.92).

#### 2.2.3 Critical Thinking Disposition

This study used a 13‐item Korean scale to measure critical thinking disposition (Ahn et al. 2009). Item examples included “I am confident in reaching a reasonable conclusion” and “I try to make decisions based on facts.” Participants were asked to rate their agreement levels with each item on a 5‐point Likert scale (1 = strongly disagree, 2 = disagree, 3 = neutral, 4 = agree, 5 = strongly agree). The average scores of the items were calculated, and higher scores represented greater levels of critical thinking disposition (Cronbach's alpha coefficient = 0.93).

#### 2.2.4 Current Smoking

For this measure, lifetime smokers, defined as those who had smoked at least one puff of a cigarette in their lifetime, were divided into two groups: (a) current non‐smokers (i.e., former smokers), who had not smoked during the past 30 days and (b) current smokers, who had smoked at least one cigarette during this period.

#### 2.2.5 Smoking Normative Beliefs

This study used two types of smoking normative beliefs: (a) smoking popularity and (b) smoking disapproval (Primack et al. 2007). Specifically, smoking popularity was assessed using four items that asked participants to rate their agreement levels with the notion that smoking is popular among celebrities (e.g., successful business people and wealthy individuals). Smoking disapproval was assessed using three items that asked participants to rate their agreement levels with parents', friends', and peers' opposition to the participants' smoking. The two normative beliefs were measured on a 4‐point Likert scale (1 = strongly disagree, 2 = disagree, 3 = agree, 4 = strongly agree). Average scores were calculated for each belief, and higher scores represented greater smoking popularity and greater smoking disapproval (Cronbach's alpha coefficients = 0.83 for smoking popularity and 0.85 for smoking disapproval).

#### 2.2.6 Demographics

A critical review of the literature identified sex, age, and weekly pocket money as key demographic factors associated with adolescent smoking (Tyas and Pederson 1998).

Among these factors, sex has been identified as a particularly important correlate of adolescent smoking in Asian contexts (Tyas and Pederson 1998). Therefore, these three factors were controlled in the psychometric evaluation to obtain more accurate findings. Sex was measured with two possible responses (male or female), and age was measured as a continuous variable. The average weekly pocket money was measured with dichotomous response options: “< 30,000 Korean won” or “≥ 30,000 Korean won.”

### 2.3 Procedures

This study obtained permission from the developers to translate the English‐language SMLS into Korean (Primack et al. 2014). The development of the K‐SMLS included three phases: (a) translation and cross‐cultural adaptation, (b) exploratory factor analysis (EFA) and confirmatory factor analysis (CFA), and (c) psychometric evaluation.

#### 2.3.1 Translation and Cross‐Cultural Adaptation

The cultural relevance of the K‐SMLS items for Korean adolescents was established through the translation and cross‐cultural adaptation processes recommended by Sousa and Rojjanasrirat (2011) and the World Health Organization (2020). First, two native Korean bilingual translators, who were knowledgeable about SML, translated the English version into Korean. Second, another native Korean bilingual translator compared the Korean version with the English version to identify ambiguities and differences in words, sentences, and meanings. Third, all three translators discussed and modified the translation to resolve identified issues and reached a consensus on the preliminary Korean version.

Fourth, the preliminary Korean version was blindly back‐translated into English by another bilingual translator, whose first language was English and who was knowledgeable in health disciplines but did not have prior knowledge about the English SMLS. Fifth, to establish the initial conceptual, semantic, and content equivalence of a pre‐final Korean version (PFKV), the back‐translator and one American nursing professor compared the instructions, items, and response format between the back‐translated and original English versions. Specifically, using the modified method to validate translation (Montoya et al. 2011; Sperber 2004), each item was rated across three dimensions—comparability of language, similarity of interpretability, and degree of understandability—on a 7‐point Likert scale ranging from 1 (extremely comparable/interpretable/understandable) to 7 (not at all comparable/interpretable/understandable). Mean scores > 3 in comparability and understandability, and mean scores > 2.5 in interpretability were considered problematic. The evaluation revealed one problematic item, “People who advertise think very carefully about the people they want to buy their product,” which exceeded the predefined interpretability criterion. Thus, this item was re‐translated into Korean and included in the PFKV.

Sixth, a panel of six experts in health sciences with expertise in smoking prevention pilot‐tested the PFKV. The test involved (a) assessing translation sufficiency for semantic, idiomatic, experiential, and conceptual equivalences with the original SMLS, (b) evaluating content validity, and (c) obtaining comments to improve translation sufficiency (Beaton et al. 2000; Ohrbach et al. 2013). Translation sufficiency for each item was measured on a 3‐point Likert scale (with 1 = inadequate to 3 = adequate), and the panel's average item scores ranged from 2.33 to 3.00. The content validity index (CVI) values for all items at both item and scale levels exceeded 0.90 (Lynn 1986; Polit and Beck 2006), indicating strong expert agreement regarding the conceptual relevance of the items for Korean adolescents. A joint review of translation sufficiency and CVI values for each item indicated that no items required removal or re‐translation (Ohrbach et al. 2013). Minor revisions were nevertheless made to the PFKV based on the panel's comments (e.g., subtle wording adjustments and changes in word order) to improve the naturalness of the Korean wording (Ohrbach et al. 2013). As a pilot test of the version refined through expert review, cognitive debriefing was conducted separately with two groups, totaling 10 middle and high school students (World Health Organization 2020). During the two focus‐group debriefing sessions, adolescents were asked to provide comments regarding the clarity of the instructions, the meaning of the questions, and the appropriateness of the response options. Through cognitive debriefing, no systematic difficulties were identified with the instructions or the response options. In addition, the adolescents provided feedback on the wording of several items, which was carefully reviewed and considered minor; thus, no further revisions were deemed necessary.

#### 2.3.2 Exploratory and Confirmatory Factor Analyses

To conduct EFA and CFA, the total sample was randomly divided into two subsamples: subsample 1 (*n* = 250) and subsample 2 (*n* = 388). This approach was necessary to confirm the factor structure identified in EFA through CFA using an independent sample and to provide a more rigorous test of its robustness (Flora and Flake 2017). Suhr (2006) and Hoyle (2000) recommend (a) at least 100 participants and five times the number of items for EFA and (b) at least 200 participants for CFA. Following these guidelines, EFA and CFA were conducted using subsample 1 and subsample 2, respectively.

After confirming that subsample 1's data were suitable for EFA, this study conducted EFA on the 22 items to explore the underlying factor structure and identify items strongly associated with a factor (Watkins 2021). Subsequently, this study assessed the reliability of the EFA‐derived scale. In the next step, using data from subsample 2, this study conducted CFA to examine whether the factor structure identified through EFA fit the data well (Acock 2013). Finally, this study assessed the reliability of the CFA‐derived scale.

#### 2.3.3 Psychometric Evaluation

Finally, this study evaluated the psychometric properties of the K‐SMLS by assessing its reliability and validity (DeVellis 2017). Reliability was assessed using internal consistency. Convergent validity was evaluated by examining the associations between the K‐SMLS and two measures (media literacy and critical thinking disposition) conceptually related to SML. Additionally, concurrent validity was evaluated by examining the relationships between the K‐SMLS and three smoking characteristics: current smoking and the two types of smoking normative beliefs.

### 2.4 Statistical Analysis

First, sample characteristics were examined using descriptive statistics. Second, the adequacy of subsample 1's data for EFA was assessed by (a) examining skewness and kurtosis and (b) conducting the Kaiser‐Meyer‐Olkin (KMO) test and the Bartlett's test for sphericity (Watkins 2021). Third, after confirming the data's adequacy, the initial EFA was conducted on the 22 items using the principal factors method. Factors were extracted based on the eigenvalue‐one criterion, the scree test, at least 10% of the common variance explained, and theoretical knowledge (O'Rourke and Hatcher 2013; Watkins 2021). Retained factors were rotated using an oblique rotation, specifically Promax, with Kaiser normalization (Field 2009; O'Rourke and Hatcher 2013), because the original scale assumed inter‐correlations among factors (Primack et al. 2014). The rotated factor pattern was reviewed for model interpretability (O'Rourke and Hatcher 2013; Watkins 2021). Following this review, items were removed to improve the factor structure based on the following criteria: factor loadings below 0.40, cross‐loadings, or communalities below 0.25 (O'Rourke and Hatcher 2013; Zeller 2005). In the next step, using the reduced items, this study again conducted the KMO test, the Bartlett's test, and EFA. Additionally, the reliability of the EFA‐derived scale and its subscales was assessed by calculating Cronbach's alpha coefficients (O'Rourke and Hatcher 2013).

Fourth, to confirm the factor structure identified through EFA, CFA with the maximum likelihood estimation method was conducted using subsample 2's data (Acock 2013). Model fit was assessed based on goodness‐of‐fit indices as well as the magnitude and statistical significance of factor loadings (O'Rourke and Hatcher 2013). Acceptable fit indices were a non‐significant chi‐square statistic, root mean squared error of approximation (RMSEA) < 0.08, comparative fit index (CFI) > 0.90, Tucker‐Lewis index (TLI) > 0.90, and standardized root mean squared residual (SRMR) < 0.08 (Acock 2013; Schumacker and Lomax 2010). Subsequently, modification indices were reviewed to identify potential improvements in model fit, and model adjustments were made based on theoretical justification (Brown 2015). Additionally, the reliability of the CFA‐derived scale and its subscales was assessed by calculating Cronbach's alpha coefficients (O'Rourke and Hatcher 2013).

Finally, the psychometric properties of the K‐SMLS were evaluated by assessing reliability and validity (DiIorio 2006; O'Rourke and Hatcher 2013). Specifically, reliability was assessed by calculating Cronbach's alpha coefficients for the K‐SMLS and its subscales in the total sample. O'Rourke and Hatcher (2013) explained that Cronbach's alpha coefficients of 0.70 or higher are acceptable. Convergent validity was assessed using Pearson's correlation coefficients to investigate the relationships with media literacy and critical thinking disposition in the total sample (*N* = 638; Field 2009). Concurrent validity was assessed by examining the relationships of SML with the three smoking characteristics. Specifically, logistic regression analysis was used to investigate the relationship between SML and current smoking (coded as 1 = current smoker and 0 = current non‐smoker) among 90 lifetime smokers, as current smoking was assessed only in this subgroup. Current smoking was used as an outcome because it is the standard measure of adolescent smoking (World Health Organization 2024) and, unlike lifetime smoking—which includes single‐puff experimentation—may more accurately reflect active smoking behavior. Two multiple linear regression analyses were then conducted to examine the relationships of SML with smoking popularity and smoking disapproval (*n* = 488 participants assessed for these variables; Field 2009). Sex, age, and weekly pocket money were adjusted for in all three regression analyses. Smoking status was additionally controlled for in regression analyses of smoking popularity and disapproval. Multicollinearity was not a problem among the independent variables in all regression analyses (Allison 1999).

## 3 Results

### 3.1 Exploratory Factor Analysis

Normality was not a problem in subsample 1's data (Fabrigar and Wegener 2012; Fitzpatrick and Wallace 2006). The KMO test and the Bartlett's test for sphericity further supported the data's suitability for EFA (Ngulube 2022), finding a KMO value above 0.85 and a statistically significant result for the Bartlett's test (*p* < 0.001). In the initial EFA, two factors were extracted based on the four criteria stated in the Statistical Analysis section. The eigenvalues of these factors were greater than one, specifically 6.46 and 2.20. The scree test further indicated that factors 1 and 2 should be retained. These factors accounted for 59.77% and 20.36% of the common variance, respectively, while each of the remaining factors contributed less than 10%. The characteristics of the two extracted factors aligned with theoretical knowledge in previous research: SSML and GML (Primack et al. 2014). After the oblique rotation of the two factors, five items were dropped from the final solution due to factor loadings below 0.40 or communalities below 0.25. In the SSML subscale, the dropped items included one item for the MM core concept and one item for the RR concept. In the GML subscale, the dropped items included two items for the AA concept and one item for the RR concept. Examples of dropped items included “Smoking scenes in movies are carefully designed” and “In general, advertisements omit a lot of important information.”

A subsequent EFA on the remaining 17 items was conducted after confirming the data's adequacy for EFA: a KMO value of 0.84 and a statistically significant result for the Bartlett's test (*p* < 0.001). The EFA identified a two‐factor solution (Table 1). Specifically, the eigenvalues of the two factors were 5.36 and 2.14. The scree test also confirmed the significance of both factors. The two factors accounted for 87.79% of the common variance and 44.12% of the total variance. Although the total variance explained was slightly lower than typically recommended, it was acceptable, given that lower proportions of the total variance explained often occur in social sciences (Hair et al. 2014). The rotated solution revealed a simple structure: Each factor had significant loadings from at least eight items, and all items loaded substantially on a single factor. These two factors corresponded to the two subscales of the original SML scale (i.e., SSML and GML; Primack et al. 2014). This study found an inter‐factor correlation of 0.37, supporting the discriminant validity of the factors and the use of the two factors as separate subscales, as suggested by the original scale developers (Brown 2015; Watkins 2021). The reliability of this scale was satisfactory: Cronbach's alpha coefficients were 0.87 for the EFA‐derived scale, 0.83 for the SSML subscale, and 0.88 for the GML subscale.

**Table 1:** Exploratory factor analysis for a korean version of the smoking media literacy scale (*n* = 250).

Item	Factor Loading		Communality
	1	2	
Smoking‐specific media literacy			
1. Tobacco companies will slyly avoid criticism and do whatever it takes for monetary profit.	0.47		0.36
2. Some cigarette brands have been designed to especially appeal to children and teenagers.	0.68		0.48
3. Cigarette advertisements associate smoking with people's innate desires, such as love, physical attractiveness, and power.	0.75		0.57
4. Wearing a shirt that portrays the logo of a cigarette brand makes you a walking billboard.	0.51		0.30
5. There are often hidden motives behind cigarette advertisements.	0.41		0.25
6. If people are exposed to cigarette advertisements, there is a strong possibility that they will start smoking.	0.65		0.37
7. If people watch movies that include smoking scenes, there is a strong possibility that they will start smoking.	0.70		0.46
8. Cigarette advertisements show scenes depicting an image of health in order to overlook the health risks.	0.58		0.32
9. Most movies and TV programs with smoking scenes make the smoking scenes appear more appealing than real life.	0.56		0.37
General media literacy			
10. Two people who watched the same movie or TV program may have significantly different thoughts about what they watched.		0.79	0.57
11. Two people who watched the same advertisement may have significantly different thoughts about what they watched.		0.80	0.57
12. Whether aware of it or not, people are influenced by TV or movies.		0.71	0.60
13. Whether aware of it or not, People are influenced by advertisements.		0.65	0.53
14. When people develop movies or TV programs, individual camera shots are carefully designed.		0.71	0.47
15. When people develop advertisements, individual camera shots are carefully designed.		0.67	0.44
16. In general, movies or TV programs do not portray life as it is.		0.57	0.45
17. When watching advertisements, it is very important to think about what was omitted.		0.51	0.41

### 3.2 Confirmatory Factor Analysis

**Figure 1:**
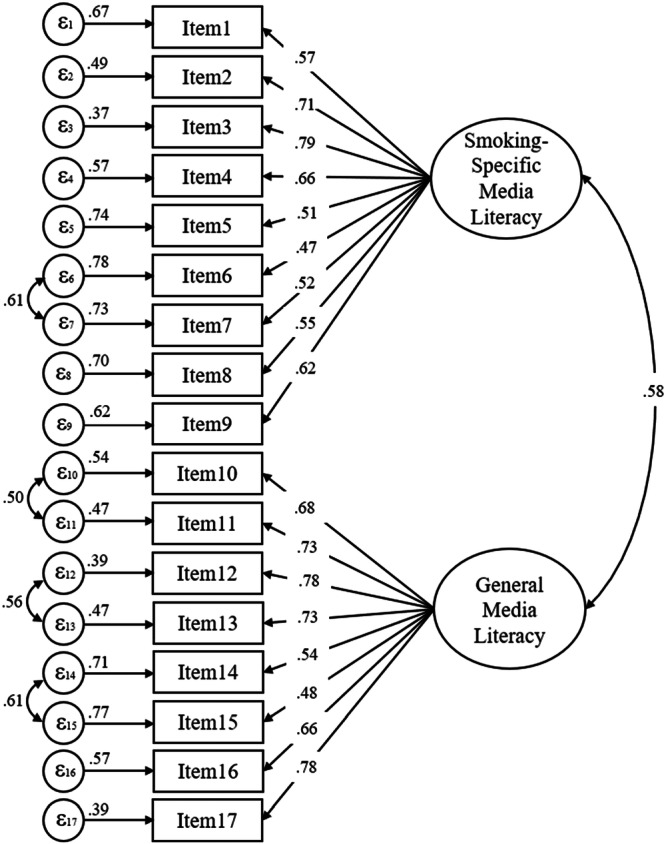
Confirmatory factor analysis for a korean version of the smoking media literacy scale (*n* = 388). *Note:* Overall model fit: χ^2^(*df*) = 250.77 (114); *p* < 0.001, RMSEA = 0.06, CFI = 0.95, TLI = 0.95, SRMR = 0.06.

In CFA conducted using subsample 2 to validate the EFA‐derived two‐factor structure, all item loadings were statistically significant and exceeded 0.40. Despite this, the model fit was not satisfactory: χ^2^(*df*) = 777.55 (118); *p* < 0.001, RMSEA = 0.12, CFI = 0.78, TLI = 0.74, SRMR = 0.08. The four largest modification indices indicated correlated error terms among the following four item pairs: items #6 and #7, #10 and #11, #12 and #13, and #14 and #15 (Table 1). Brown (2015) explained that uncorrelated error terms can be a significant source of inadequate model fit, particularly among similarly worded items in self‐report questionnaires. The model was revised to include error‐term correlations for each of the four pairs of similarly worded items (Figure 1). The revised model fit the data well, as evidenced by the following fit statistics: χ^2^(*df*) = 250.77 (114); *p* < 0.001, RMSEA = 0.06, CFI = 0.95, TLI = 0.95, SRMR = 0.06. Notably, the significant chi‐square statistic was not problematic because it often appears significant in well‐fitting models (O'Rourke and Hatcher 2013). An inter‐factor correlation of 0.58 in the revised model confirmed the discriminant validity of the two factors (Brown 2015; Watkins 2021). The reliability was satisfactory: Cronbach's alpha coefficients were 0.88 for the CFA‐derived scale, 0.84 for the SSML subscale, and 0.88 for the GML subscale.

### 3.3 Psychometric Properties

This study found satisfactory psychometric properties for the K‐SMLS derived through EFA and CFA. First, the reliability was satisfactory: Cronbach's alpha coefficients were 0.88 for the K‐SMLS, 0.84 for the SSML subscale, and 0.88 for the GML subscale. Second, this study found strong evidence supporting convergent and concurrent validity (Tables 2 and 3). For convergent validity, the K‐SMLS had positive, moderate, statistically significant correlations with media literacy (*r* = 0.39) and critical thinking disposition (*r* = 0.34). These correlation coefficients served as satisfactory evidence for convergent validity, because correlations between conceptually related but theoretically non‐identical constructs are expected to be positive yet moderate rather than extremely high (Bartolucci et al. 2015; DeVellis 2017; Wilson and Joye 2016). For concurrent validity, a one‐point increase in the K‐SMLS was significantly associated with a 33% reduction in the risk of current smoking (odds ratio = 0.67), after adjusting for demographics. Additionally, the K‐SMLS was strongly associated with two types of smoking normative beliefs. Specifically, after controlling for demographics and current smoking status, a one‐point increase in the K‐SMLS was significantly associated with a 0.40‐point decrease in the popularity of smoking (regression coefficient = −0.40) and a 0.05‐point increase in smoking disapproval by parents and peers (regression coefficient = 0.05).

**Table 2:** Pearson correlation coefficients and descriptive statistics for K‐SMLS, media literacy, and critical thinking disposition (*N* = 638).

Measure	Pearson correlation coefficient (*p*‐value)			Mean	Standard deviation	Score range
	1	2	3			
1. K‐SMLS	—			6.41	1.48	0.00–10.00
2. Media Literacy	0.39 (< 0.001)	—		3.75	0.56	2.06–5.00
3. Critical thinking disposition^a^	0.34 (< 0.001)	0.61 (< 0. 001)	—	3.69	0.62	1.00–5.00

Abbreviation: K‐SMLS, Korean version of the smoking media literacy scale.

^a^ Asked and measured among 488 participants.

**Table 3:** Relationships of K‐SMLS with current smoking and smoking normative beliefs.

Variable	Current smoking (*n *= 90)				Smoking normative belief (*n* = 488)							
					Popularity of smoking among the successful and elite				Smoking disapproval by parents and peers			
	OR	95% CI		*p*‐value	B	S.E.	*β*	*p*‐value	B	S.E.	*β*	*p*‐value
		Lower	Upper									
K‐SMLS	0.67	0.45	0.99	0.045	−0.40	0.02	−0.09	0.041	0.05	0.03	0.10	0.031
Control variable												
Sex (ref.: male)												
Female	0.82	0.30	2.30	0.711	−0.03	0.06	−0.03	0.545	0.36	0.07	0.23	< 0.001
Age	1.14	0.76	1.70	0.523	0.02	0.02	0.07	0.160	−0.05	0.02	−0.11	0.027
Average weekly pocket money (ref.: < 30,000 Korean Won)												
≥ 30,000 Korean Won	3.21	1.20	8.57	0.020	0.14	0.06	0.11	0.021	−0.17	0.08	−0.10	0.032
Smoking status (ref.: lifetime non‐smoking)												
Former smoking	—	—	—	—	0.37	0.11	0.15	0.001	−0.37	0.14	−0.12	0.010
Current smoking	—	—	—	—	0.46	0.10	0.21	< 0.001	−0.22	0.13	−0.80	0.087

Abbreviations: B, regression coefficient; CI, confidence interval; K‐SMLS, Korean version of the smoking media literacy scale; OR, odds ratio; ref, reference group; S.E., standard error; *β*, standardized regression coefficient.

## 4 Discussion

Through the processes of translation, cultural adaptation, and validation, this study established the 17‐item, two‐factor K‐SMLS based on the original 22‐item English SMLS (Primack et al. 2014). The two factors identified in the K‐SMLS were consistent with the theoretical two‐factor structure of the original scale: SSML and GML (Primack et al. 2014; Primack et al. 2006). Empirical research validating the original SMLS is scarce in the existing literature, which makes the present findings difficult to compare with previous studies. Although one three‐factor SML scale has been developed for Korean adolescents, it was based on a different version of the SMLS, differs in item composition, and has several methodological limitations, as stated in the Introduction section (Kim et al. 2021). Additionally, one study conducted translation, back‐translation, and principal component analysis (PCA) on a 21‐item version of the original SMLS, and identified a three‐factor structure (Page, Piko, et al. 2011), which differed from the theoretical structure proposed by the original scale developers (Primack et al. 2014; Primack et al. 2006). However, caution is required in interpreting these results because the primary purpose of the study by Page, Piko, et al. (2011) was to examine the associations between SML and smoking characteristics, and it had major analytical limitations (e.g., the use of PCA and a lack of validity testing). Furthermore, it did not report (a) specific details of the translation, back‐translation, and PCA processes and (b) related findings.

Through EFA in this study, two items were removed from the SSML subscale, and the remaining items encompassed all three core concepts of media literacy. In the GML subscale, three items were removed, and the remaining items covered two core concepts (MM and RR). The removal of these items from the K‐SMLS might be partly attributed to potential issues of item clarity and the inherent complexity of the core concepts of media literacy (DeVellis 2017; Ptaszek 2019; Schilder et al. 2016). Specifically, items that lacked clarity may have been misinterpreted by respondents, which could result in unreliable measurement of the intended core concepts (DeVellis 2017). In addition, while the literature has consistently indicated that the core concepts of media literacy possess overlapping attributes (Hobbs n.d.; Moses 2021), the precise nature of their inter‐relationships remains empirically underexplored (Hobbs 2006; Primack et al. 2006). For example, both very low and excessively high correlations among AA, MM, and RR concepts are problematic for their measurement as distinct core concepts. Low correlations suggest that core concepts may not measure the same construct (Watkins 2021), while high correlations indicate excessive redundancy, which compromises the discriminant validity of the core concepts (DeVellis 2017).

The K‐SMLS demonstrated satisfactory psychometric properties. It exhibited excellent reliability: For the K‐SMLS and its two subscales, Cronbach's alpha coefficients were within the ideal range of 0.80 to 0.90 (O'Rourke and Hatcher 2013). Additionally, the K‐SMLS showed satisfactory convergent and concurrent validity. For convergent validity, the K‐SMLS had positive, moderate, statistically significant correlations with media literacy and critical thinking disposition. These findings aligned with the literature: (a) Media literacy is an umbrella concept encompassing sub‐literacies focusing on specific media practices, and thus considerable overlap exists among them (Considine et al. 2009); and (b) Cognitive skills related to critical thinking are required to enhance media literacy levels (Brown 1998; Brown 2006). For concurrent validity, the K‐SMLS was negatively associated with the risk of current smoking and two pro‐smoking normative beliefs. These findings were consistent with the literature, demonstrating that higher SML levels were associated with lower levels of smoking risk, pro‐smoking norms, and positive attitudes toward smoking (Bier et al. 2016; Chang et al. 2016; Page, Huong et al. 2011; Page, Piko et al. 2011; Primack et al. 2006; Salgado et al. 2012).

### 4.1 Strengths and Limitations of the Study

The primary strength of this study was its contribution to addressing a significant gap in the psychometric evaluation of SML measures in the current literature (Huguet et al. 2021; Ptaszek 2019). This study used more refined research methodologies to obtain more accurate findings than previous studies (Kim et al. 2021; Page, Piko et al. 2011). For example, despite using a convenience sampling method, this study maximized sample diversity by recruiting participants from nine schools across multiple districts. The sample was also heterogeneous across sex, school year, and school type. In addition, this study employed a comprehensive and rigorous methodological approach (e.g., expert content validation, EFA and CFA with separate samples, and multiple forms of validity testing).

Despite the strengths of the study, the current findings should be interpreted with caution in light of three limitations. First, caution is needed when generalizing the study findings to the population of Korean adolescents due to the use of a convenience sample (Reynolds and Guest 2015). Second, predictive validity and test‐retest reliability could not be assessed to psychometrically evaluate the K‐SMLS due to the cross‐sectional design of the study (DeVellis 2017). Third, the use of self‐report measures might have led to measurement errors due to social desirability bias (Hageman et al. 2015). For example, participants might have underreported smoking behaviors to avoid social stigma.

## 5 Conclusion

This study established the K‐SMLS through the processes of translation, cultural adaptation, and validation. This study could contribute to (a) addressing a significant knowledge gap in the psychometric evaluation of SML scales and (b) facilitating efforts to protect Korean adolescents from pro‐smoking messages. Despite these advancements, future studies should continue to psychometrically evaluate SML scales to resolve current issues in this research field, such as the limited knowledge of these scales' cross‐cultural applicability, insufficient empirical testing of theoretical frameworks, and the absence of comparable and standardized scales (Hobbs n.d.; Ptaszek 2019; Schilder et al. 2016). To address these issues, future empirical studies should use (a) culturally diverse samples (Huguet et al. 2021), (b) items that accurately measure core concepts of SML based on more refined theoretical frameworks (Huguet et al. 2021), (c) representative samples to enhance the generalizability of study findings (Adams and McGuire 2022), (d) longitudinal study designs to assess additional psychometric properties, such as predictive validity and test‐retest reliability (DeVellis 2017), and (e) strategies that further ensure the anonymity of responses to reduce social desirability bias (Adams and McGuire 2022).

### 5.1 How Might This Information Affect Nursing Practice?

The K‐SMLS, which was developed through rigorous psychometric evaluation, has potential practical implications for nurses. By using this scale, nurses may more precisely assess SML levels among Korean adolescents, identify those who are more susceptible to pro‐smoking messages, and provide targeted education (Ptaszek 2019). In addition, the use of the K‐SMLS may contribute to addressing the current issue of incomparability across study findings due to the absence of validated instruments in this population (Huguet et al. 2021; Ptaszek 2019). Specifically, the understanding of SML‐related phenomena may be further enhanced through empirical studies that use this scale to compare intervention effects and generate evidence‐based conclusions (Huguet et al. 2021). Ultimately, more accurate empirical evidence may contribute to the development of targeted strategies to enhance adolescent SML (Bulger 2012; Huguet et al. 2021).

## Ethics Statement

This study was approved by the Human Research Ethics committee of Kyung Hee University (Ethics approval number: KHSIRB‐17‐066‐1[RA]).

## Conflicts of Interest

The author declares no conflicts of interest.

## Data Availability

The data that support the findings of this study are available from the corresponding author upon reasonable request.
